# Antipsychotics, mood stabilisers, and risk of violent crime

**DOI:** 10.1016/S0140-6736(14)60379-2

**Published:** 2014-09-27

**Authors:** Seena Fazel, Johan Zetterqvist, Henrik Larsson, Niklas Långström, Paul Lichtenstein

**Affiliations:** a Department of Psychiatry, University of Oxford, Warneford Hospital, Oxford, UK; b Department of Medical Epidemiology and Biostatistics, Karolinska Institutet, Stockholm, Sweden

## Abstract

**Background:**

Antipsychotics and mood stabilisers are prescribed widely to patients with psychiatric disorders worldwide. Despite clear evidence for their efficacy in relapse prevention and symptom relief, their effect on some adverse outcomes, including the perpetration of violent crime, is unclear. We aimed to establish the effect of antipsychotics and mood stabilisers on the rate of violent crime committed by patients with psychiatric disorders in Sweden.

**Methods:**

We used linked Swedish national registers to study 82 647 patients who were prescribed antipsychotics or mood stabilisers, their psychiatric diagnoses, and subsequent criminal convictions in 2006–09. We did within-individual analyses to compare the rate of violent criminality during the time that patients were prescribed these medications versus the rate for the same patients while they were not receiving the drugs to adjust for all confounders that remained constant within each participant during follow-up. The primary outcome was the occurrence of violent crime, according to Sweden's national crime register.

**Findings:**

In 2006–09, 40 937 men in Sweden were prescribed antipsychotics or mood stabilisers, of whom 2657 (6·5%) were convicted of a violent crime during the study period. In the same period, 41 710 women were prescribed these drugs, of whom 604 (1·4 %) had convictions for violent crime. Compared with periods when participants were not on medication, violent crime fell by 45% in patients receiving antipsychotics (hazard ratio [HR] 0·55, 95% CI 0·47–0·64) and by 24% in patients prescribed mood stabilisers (0·76, 0·62–0·93). However, we identified potentially important differences by diagnosis—mood stabilisers were associated with a reduced rate of violent crime only in patients with bipolar disorder. The rate of violence reduction for antipsychotics remained between 22% and 29% in sensitivity analyses that used different outcomes (any crime, drug-related crime, less severe crime, and violent arrest), and was stronger in patients who were prescribed higher drug doses than in those prescribed low doses. Notable reductions in violent crime were also recorded for depot medication (HR adjusted for concomitant oral medications 0·60, 95% CI 0·39–0·92).

**Interpretation:**

In addition to relapse prevention and psychiatric symptom relief, the benefits of antipsychotics and mood stabilisers might also include reductions in the rates of violent crime. The potential effects of these drugs on violence and crime should be taken into account when treatment options for patients with psychiatric disorders are being considered.

**Funding:**

The Wellcome Trust, the Swedish Prison and Probation Service, the Swedish Research Council, and the Swedish Research Council for Health, Working Life and Welfare.

## Introduction

Antipsychotic drugs and mood stabilisers are widely prescribed for a range of psychiatric disorders, including schizophrenia and related disorders, bipolar disorder, severe depression, and other diagnoses. In 2007, an estimated 3·9 million Americans (1·3% of the population) purchased antipsychotics at a total cost of US$7·4 billion, which represents a three-fold increase within a decade.^1^ A similar pattern exists in other high-income countries:^2^, ^3^ antipsychotic prescriptions in the UK increased by 82% between 1998 and 2010 (a 5·1% rise per year),^4^ and the number of such prescriptions tripled in Australia between 2000 and 2011.^5^ Large increases in prescriptions for mood stabilisers have also been recorded,^5^, ^6^ with 0·3–0·4% of people in the USA prescribed these drugs in 2007.^7^ Systematic reviews of trial data have shown that antipsychotics and mood stabilisers have beneficial effects on relapse and readmission rates in schizophrenia,^8^ bipolar disorder,^9^, ^10^ treatment-resistant depression,^11^ and borderline personality disorder.^12^ However, evidence about the effects of pharmacotherapy on other important outcomes,^13^, ^14^ including violent behaviour,^15^ is scarce.

The perpetration of interpersonal violence and its consequences are among the most important adverse outcomes for patients with psychiatric disorders.^16^ Recent reviews suggest that the relative risk of violence against other people is four-times higher in patients with schizophrenia and related psychoses than in the general population,^17^ and estimated absolute rates of violence are 28% within 1 year of discharge from US inner city hospitals^18^ and 5% in patients who are not admitted to hospital.^19^ In patients with bipolar disorder, rates of violence are substantially increased in cases of substance misuse.^20^

Consequently, the reduction of violence risk in psychiatric patients is a core component of clinical care, and clinical guidelines in the USA and UK recommend risk assessment of violence in patients with schizophrenia.^21^, ^22^ However, evidence for effective therapeutic approaches to manage the risk of violence is not strong, has mostly been generalised from offenders without mental disorders, and is focused on psychological interventions.^23^ According to reviews^24^, ^25^ and clinical guidelines,^26^ the existing evidence base for pharmacological strategies to reduce violence risk is weak or inconclusive. Furthermore, although depot injection of antipsychotics seems to reduce relapse rates further compared with orally administered antipsychotics,^27^, ^28^ whether or not such benefits extend to reductions in violence risk is unknown. Randomised clinical trials of drugs in which violence is investigated are scarce because low outcome rates mean that such trials would have to be unfeasibly large to show differences between the intervention and control groups. Furthermore, aggressive or hostile patients are less likely to be recruited, consent, or remain in the study than are patients without such traits, and such trials could be difficult to justify ethically because of the immediate need for treatment in some patients. Pharmacoepidemiological approaches offer an alternative in that they compare rates of violence in patients taking antipsychotics with those in people who are not. However, this design is limited by confounding by indication—the patients taking such drugs have different background risk factors for violence to those who are not on medication. Propensity scoring attempts to adjust for this; nevertheless, residual confounding is likely to bias results.^29^ This limitation is partly addressed by within-individual designs, in which rates of violence when patients are on medication are compared with the rates when they are not taking medication. This design accounts for confounders that remain stable within the same patient, although such studies cannot prove causality because other time-varying factors could be associated with adherence to treatment.

Therefore, we undertook a national pharmacoepidemiological study using data from high-quality nationwide registers during 2005–09, in which we did within-individual analyses. We tested four hypotheses: antipsychotics and mood stabilisers reduce rates of violent offending in patients prescribed these medications; rate reductions differ according to major diagnostic categories; combination treatment with antipsychotics and mood stabilisers further improves violent outcomes; and depot antipsychotics reduce the risk of violence by a greater extent than do oral antipsychotics.

## Methods

### Study design and patients

We obtained data for this study through linkage of national longitudinal population-based registers in Sweden; unique personal identification numbers enabled accurate data linkage across registers.^30^ We initially included all people in Sweden born between Jan 1, 1961, and Dec 31, 1990 (1 944 548 men and 1 858 984 women), so that all participants were at least 15 years of age (the Swedish age of criminal responsibility) at the start of follow-up in July, 2005. From this population, we identified our primary sample: people prescribed mood stabilisers or antipsychotics according to the Swedish Prescribed Drug Register. This register includes information about all prescribed and dispensed medication since July, 2005, including the exact dates of the dispensed prescription,^31^ and is reported to be complete, with less than 0·3% of entries having missing patient identity data.^31^ We identified violent criminality through the national crime register, which includes convictions in all Swedish district courts since 1973.^32^ For sensitivity analyses, we also used the register of persons suspected of offences, which includes people who are suspected of crime after a completed investigation by police, customs authority, or prosecution service.^32^ The crime register has excellent coverage: in a 13-year study, only 0·05% of cases had incomplete personal identifiers.^33^ We also identified emigrations and deaths by linking individuals to the migration and cause of death registers, so that the actual time at risk for crime outcomes could be accounted for. Periods in prison were accounted for by linkage to the prison register, and times spent in psychiatric hospitals were estimated with the National Patient Register, which includes data for all psychiatric hospital admissions since 1973 (and for outpatient care since 2001).^34^

The study was approved by the Regional Ethics Committee at Karolinska Institutet, Sweden.

### Definitions and measures

We extracted data about treatment with antipsychotics and mood stabilisers, identified in the Swedish Prescribed Drug Register according to the Anatomical Therapeutic Chemical (ATC) classification system. Antipsychotics were defined as drugs with ATC codes N05A, excluding lithium (N05AN01) and clozapine (N05AH02). Mood stabilisers were defined as valproic acid or sodium valproate (N03AG01), lamotrigine (N03AX09), carbamazepine (N03AF01), oxcarbazepine (N03AF02), and lithium. Clozapine was coded separately because it is licensed only for patients with treatment-resistant psychosis (which has shown inadequate response to treatment in two adequate trials—ie, those of 4 weeks at optimum dose—of other antipsychotics)^26^ and necessitates regular blood testing, which suggests that patients who are prescribed this drug are a selected group. Furthermore, clozapine's efficacy is significantly higher than that of other antipsychotics.^35^ Antipsychotic depot preparations were identified as injections administered every 2 weeks or longer.

We defined start of treatment as the date of the first prescription, and end of treatment as the date of the final prescription during the study period. These dates are the days on which the prescriptions were collected. A patient was defined as receiving treatment during the time interval between two dispensed prescriptions of medication, unless prescriptions were issued more than 4 months apart. We chose this time interval because in routine psychiatric practice, oral medications are unlikely to be dispensed for more than 3 months at a time (the so-called 90-day rule in Sweden). Thus, a treatment period was defined as a sequence of at least two prescriptions, with no more than 4 months between any two consecutive prescriptions. During periods of more than 4 months without any new prescription, the patient was judged to be off treatment. However, for antipsychotic depots, which are administered not by patients but by health-care staff, we allowed an interval of up to 1 year between two consecutive prescriptions in a treatment period. Patients who received a prescription only once (n=20 700) were judged to be off treatment throughout the study period, and did not contribute to our within-individual estimates. To establish whether participants were receiving treatment at the start and end of follow-up, we needed information about prescription dates in the 4 months before and the 4 months after follow-up. Since the Prescribed Drug Register covered the period July 1, 2005–June 30, 2010, the start of follow-up was set as Jan 1, 2006 to avoid any selection bias with an earlier date. We had register information until Dec 31, 2009, which was set as end of follow-up.

We also used defined daily doses to analyse the effects of medication dose, in which we compared the effects of 0, 1, and 2 defined daily doses on conviction for violence (appendix).

### Outcomes

The primary outcome was conviction for a violent crime—defined as any criminal conviction for homicide, assault, robbery, arson, any sexual offence, illegal threats, or intimidation^36^—according to data from the national crime register. We did not include suspected violent crime in the main analyses. We used the date of the committed crime; for cases in which a time window was given for the date of the crime, we used the earliest date. For 77% of the crimes, we could establish the date with an accuracy of 1 week. We excluded cases for which no specific crime date had been recorded (119 violent convictions [1·17%]) because conviction dates can be much later than crime dates and might distort the correct ordering of events.

### Diagnostic categories

We used the National Patient Register to identify patients diagnosed with schizophrenia, bipolar disorder, other psychotic disorders (apart from schizophrenia and bipolar disorder), and depression. In accordance with the International Classification of Diseases tenth revision diagnostic guidelines, we used a hierarchical diagnostic system—patients with schizophrenia at any time were diagnosed with schizophrenia, followed by bipolar disorder, and then other psychoses. This approach has also been used in previous studies (see appendix for diagnostic codes).^37^ We included depression because mood stabilisers and antipsychotics are used frequently in its treatment. The diagnostic validity is strong for schizophrenia (concordance rates of 86% in comparisons with file reviews by psychiatrists)^38^ and bipolar disorder (concordance of 92%).^39^

### Statistical analyses

Initially, we did between-individuals analyses, which included all people who had been on medication at some point during the 4 years of the study, and we compared violent crime rates in patients on medication with those who were not on medication. Medication groups were combined into one time-dependent binary covariate, with one level for any medication and another for no medication. To account for the correlations between periods within the same patient, we calculated robust standard errors. We adjusted the between-individual analysis for sex and age (as a time-dependent covariate).

However, our main approach was to undertake within-individual analyses. We did these analyses with stratified Cox regression—we entered each patient as a separate stratum in the analysis, which adjusts for all confounders that remain constant within each person during follow-up. Three medication groups (antipsychotics, mood stabilisers, and clozapine) were coded as separate time-dependent covariates in the same model. For each patient, we compared the crime rate while on medication with the crime rate while off treatment for each medication group, with adjustment for the other two medication groups. We also used a similar model in which all drugs were combined into one time-dependent binary covariate. We did not adjust for age in within-individual analyses because this approach can provide erroneous estimates.^40^ Furthermore, the risk of confounding by age was judged to be low because the maximum length of follow-up was 4 years in a cohort with an average age of 31 years at the start of follow-up. More details about the statistical methods used are available in a related publication.^41^

### Sensitivity analyses

To establish whether the reported associations could be explained by selection effects and to test the robustness of our findings, we investigated different outcomes, which included any crime, less severe crime (defined as crimes not leading to custodial sentences), and drug-related crime. Additionally, we used the national register of persons suspected of offences (or suspicions register), which will have included those people who were subsequently convicted. In Sweden, people are convicted of crimes irrespective of their mental disorder, although their sentencing will be affected by psychiatric evidence. Nevertheless, the probability of being convicted could be affected by socioeconomic conditions, living area, age, or psychiatric history.

Additionally, within the cohort of patients prescribed antipsychotics or mood stabilisers, we studied the effects of being prescribed a selective serotonin reuptake inhibitor antidepressant on violent crime. This analysis was one approach to handle the possible non-specific effects of medication prescription on offending, such as regular reviews by health-care staff and links to other medical and social services. In other sensitivity analyses, we assessed the effects of a history of violent crime (before Jan 1, 2006), dose of medication, timing of medication, censoring of periods of hospital stay, age at start of follow-up, and possible adherence effects (see appendix for details).

### Role of the funding source

The funders of the study had no role in the design and conduct of the study, data gathering, management, analysis, and interpretation; or in the preparation, review, or approval of the report. JZ had full access to all the data in the study and, with SF, takes responsibility for the integrity of the data and the accuracy of the data analysis.

## Results

Of the 1 944 548 men and 1 858 984 women born in Sweden between 1961 and 1990, we identified 40 937 men and 41 710 women who were prescribed any antipsychotic or mood stabiliser between Jan 1, 2006, and Dec 31, 2009, Thus, 2·1% of the men and 2·2% of the women had been prescribed at least one of these drugs. Table 1 shows the baseline characteristics of these patients. The average age of the sample population at start of follow-up was 31·7 years for men and 31·3 years for women. During the study period, 2657 men were convicted of 4166 violent crimes, and 604 women convicted of 782 violent crimes, in this cohort.

**Table 1:** Background characteristics of patients prescribed antipsychotics and mood stabilisers in Sweden, 2006–09

			**Men (n=40 937)**	**Women (n=41 710)**
Person-years at risk			159 501	163 926
Age group (years)				
	15–24		9979 (24%)	11 105 (27%)
	25–39		21 269 (52%)	21 151 (51%)
	≥40		9689 (24%)	9454 (23%)
Civil status				
	Unknown		722 (2%)	751 (2%)
	Unmarried		31 937 (78%)	27 584 (66%)
	Married		5637 (14%)	8765 (21%)
	Divorced		2638 (6%)	4610 (11%)
	Widowed		3 (<1%)	0
Living in metropolitan area			8123 (20%)	7878 (19%)
Employed			14 636 (36%)	15 550 (37%)
Studying			4828 (12%)	7598 (18%)
Median family-adjusted income in 2006 US$			15 497 (12 434)	14 190 (10 868)
Medications taken in 2006				
	Mood stabilisers		14 753 (36%)	15 528 (37%)
	Antipsychotics		15 757 (38%)	14 283 (34%)
	Clozapine		1360 (3%)	818 (2%)
	Antidepressants (N06A)		14 420 (35%)	18 926 (45%)
	Hypnotics/anxiolytics (N05B, N05C)		16 431 (40%)	19 400 (47%)
	Stimulants (N06BA)		959 (2%)	679 (2%)
	Drug used in addictive disorders (N07B)		1931 (5%)	1125 (3%)
Psychiatric diagnosis				
	Any psychotic disorder		17 532 (43%)	16 646 (40%)
		Schizophrenia	6015 (15%)	3110 (7%)
		Bipolar disorder	4303 (11%)	7615 (18%)
		Other psychotic disorder	7214 (18%)	5921 (14%)
	Depression		5731 (14%)	8563 (21%)
	Antisocial personality disorder		588 (1%)	196 (<1%)
	Other personality disorder (except antisocial personality disorder)		5517 (13%)	8535 (20%)
	Alcohol misuse		6707 (16%)	4771 (11%)
	Drug misuse		7367 (18%)	5632 (14%)

Data are n (%) or median (SD).

First, we did between-individual analyses, in which we compared rates of violent crimes during periods on medication compared with periods not on medication in a cohort of 82 647 patients who had at least one period on medication during follow-up. In a Cox regression model, the violent crime rate was reduced by an estimated 64% for any antipsychotic or mood stabilisers (hazard ratio [HR] 0·36, 95% CI 0·34–0·39; table 2). When we made adjustments for other classes of drugs, we also noted specific reductions in violent crime for mood stabilisers, antipsychotics (apart from clozapine), and clozapine (table 2; see appendix p 9 for extended Kaplan-Meier graphs).

**Table 2:** Hazard ratios for the association between psychotropic medication and violent crime in a Swedish population cohort of 82 647 patients with prescriptions

	**Any of the three drug types**	**Mood stabiliser**	**Antipsychotic**	**Clozapine**
Within-individual*	0·57 (0·50–0·65)	0·76 (0·62–0·93)	0·55 (0·47–0·64)	0·53 (0·16–1·74)
Between-individual†	0·36 (0·34–0·39)	0·32 (0·28–0·35)	0·60 (0·54–0·65)	0·10 (0·05–0·19)

Data are hazard ratio (95% CI). In total, 4948 convicted violent crimes were committed.

* The within-individual analyses are adjusted by other psychotropic medications (mood stabilisers, antipsychotics, or clozapine).

† The between-individual analyses are adjusted by age, sex, and concomitant use of other psychotropic medications.

To account for confounders that are constant within each patient during follow-up, we did within-individual analyses to compare rates of violent crime in the same individual when they were both on and off medication (table 2). We noted substantially lower rates of violent crime when any of the three classes of medication had been prescribed, specifically for antipsychotics and mood stabilisers (table 2). Clozapine was also associated with a reduced rate of violent crime (table 2), although this decrease was not significant because of the small number of patients (2178) who received this drug. These hazard ratios did not change substantially when we censored for periods of hospital stay (appendix p 3).

In our within-individual analyses, we noted that prescription of medication was associated with similar reductions in any crime, drug-related crime, less severe crime, and suspected violent crime, including significant reductions associated with clozapine for all outcomes except drug-related crime (table 3). Second, differences in rates of violent crime were negligible between patients with previous violent convictions (9382 patients) and those without such convictions (73 261 patients) (appendix p 4). In patients with psychotic disorders, statistical power was low to test for violent crime, but we noted a trend towards larger reductions in rates of violent crime in those without violent criminal histories (appendix p 4). Third, we recorded little difference in the violent crime risk depending on the order of medication. Patients who received treatment and then discontinued had similar odds of committing violent crime to those who started their medication after a period of non-treatment (odds ratio [OR] 0·75, 95% CI 0·59–0·95 *vs* OR 0·74, 95% CI 0·59–0·94). No apparent differences were recorded by age for antipsychotics, although a trend towards stronger associations with mood stabilisers in patients older than 40 years should be investigated further (appendix p 3). Finally, we recorded stronger associations between medication and violent crime reduction with higher doses of prescribed antipsychotics (p=0·019), but this association was not significant for mood stabilisers (p=0·127) or clozapine (p=0·255) (appendix p 5). To address adherence to treatment, we recorded no differences in the rate of violence within the first 45 days after the start of treatment (short-term effect) compared with that after 45 days (long-term effect; appendix p 6).

**Table 3:** Hazard ratios for different crime outcomes in 82 647 patients prescribed mood stabilisers, antipsychotics, and clozapine, compared with periods when these same patients are not on medication (within-individual analyses)

	**Crimes in study cohort (n)**	**Mood stabiliser**	**Antipsychotic**	**Clozapine**
Any crime (convictions)	29 496	0·83 (0·77–0·90)	0·78 (0·74–0·83)	0·53 (0·33–0·86)
Drug-related crime (convictions)	10 389	0·68 (0·58–0·79)	0·71 (0·65–0·79)	0·41 (0·15–1·13)
Less severe crimes* (convictions)	23 801	0·81 (0·74–0·88)	0·77 (0·73–0·82)	0·55 (0·32–0·97)
Violent crime (suspicions†)	16 069	0·87 (0·78–0·96)	0·74 (0·68–0·79)	0·43 (0·26–0·72)

Data are n or hazard ratio (95% CI).

* Less severe crime were those that did not lead to custodial sentences.

† Suspicions were crimes that led to an arrest and preliminary investigation.

We calculated within-individual estimates for diagnostic subgroups. Rates of violent crime were significantly reduced in both male and female patients taking antipsychotics (excluding clozapine) who had diagnoses of schizophrenia, bipolar disorder, and other psychoses (table 4; see appendix p 7 for results by sex). For mood stabilisers, there was a significant reduction in violent crime in bipolar disorder in men but not in women (table 4, appendix p 7).

**Table 4:** Hazard ratios for violent crime in patients with different psychiatric disorders who have been prescribed antipsychotics and mood stabilisers (within-individual analyses)

		**Individuals in cohort (n)**	**Crimes in cohort (n)**	**Antipsychotic**	**Mood stabiliser**
Any psychotic disorder*		34 178	2445	0·50 (0·41–0·61)	0·65 (0·48–0·89)
	Schizophrenia	9 125	542	0·65 (0·45–0·93)	1·17 (0·51–2·71)
	Bipolar disorder	11 918	494	0·52 (0·29–0·92)	0·44 (0·28–0·70)
	Other psychotic disorders†	13 135	1409	0·43 (0·33–0·55)	0·91 (0·54–1·53)
Depression		14 294	848	0·77 (0·51–1·15)	0·91 (0·55–1·52)

Data are n or hazard ratio (95% CI). All analyses are adjusted for clozapine medication. Analyses were underpowered to investigate clozapine.

* Includes schizophrenia, bipolar disorder, and other psychotic disorders.

† Excludes both schizophrenia and bipolar disorder.

In our analysis of combination treatment, for which we used within-individual and between-individual approaches, we noted a significant decrease in the rate of violent crime when an antipsychotic was added to a mood stabiliser but not when a mood stabiliser was added to an antipsychotic (table 5; see appendix p 8 for results by sex).

**Table 5:** Hazard ratios for the association between violent crime and different exposures to psychotropic medications

	**Patient group**	**Number of individuals (number of crimes)**	**Within-individual estimates**	**Between-individual estimates**
Addition of an antipsychotic during periods on mood stabilisers	Patients ever prescribed both mood stabiliser and antipsychotic during follow-up	11 654 (228)	0·27 (0·09–0·79)	0·69 (0·50–0·96)
Addition of a mood stabiliser during periods on antipsychotics	Patients ever prescribed both mood stabiliser and antipsychotic during follow-up	11 654 (219)	0·77 (0·32–1·85)	0·74 (0·55–1·01)
Antipsychotic depot	Prescribed depot antipsychotic during follow-up	4904 (1162)	0·67 (0·45–1·01)	0·38 (0·32–0·54)
Antipsychotic depot*	Prescribed depot antipsychotic during follow-up	4904 (1162)	0·60 (0·39–0·92)	0·30 (0·23–0·39)
Antipsychotic oral medication	Prescribed oral antipsychotic during follow-up	47 235 (3864)	0·53 (0·45–0·62)	0·42 (0·38–0·46)
SSRI medication	Prescribed mood stabiliser, antipsychotic, or clozapine during follow-up	82 647 (4948)	1·15 (0·96–1·37)	0·95 (0·86–1·06)

Data are n or hazard ratio (95% CI). SSRI=selective serotonin reuptake inhibitor.

* Adjusted for concomitant use of oral antipsychotics, mood stabilisers, and clozapine.

To investigate depot antipsychotics, we separated these drugs from oral antipsychotics and restricted the cohort to patients with at least one prescription of depot antipsychotics during follow-up (table 5, and appendix p 8 by sex). In within-individual analyses, when adjusting for oral antipsychotics and mood stabilisers, we noted a significant reduction in violence overall (table 5).

Finally, we assessed the possible effects of selective serotonin reuptake inhibitor antidepressants within the group of patients prescribed antipsychotics, mood stabilisers, or both. Here, when we compared rates of violent crime convictions when individuals were on selective serotonin reuptake inhibitors compared with when these same individuals were not (irrespective of changes to their other medication), these rates did not differ overall (table 5).

## Discussion

In this study of 82 627 patients prescribed antipsychotics and mood stabilisers over 4 years, we recorded an association between antipsychotic drugs and reductions in the rate of violent crime in the same people when they were on medication compared with when they were not. Importantly, our approach accounted for confounding factors that remained stable within the same patient. Our series of sensitivity analyses suggested similar rate reductions when any crime, less severe crime, drug-related crime, and arrests on suspicion of violent crime were used as alternative outcomes, and in people without a history of violent crime. In addition to the apparently protective effect of antipsychotic medication, we recorded similar findings for mood stabilisers, especially in patients with bipolar disorder. Although a direct causal interpretation is not possible, these associations might have important implications for clinical practice. The first and main such implication is that antipsychotics could have beneficial effects on violent crime outcomes. Our results are consistent with a recent synthesis of trial data of five trials with 403 participants that suggested absolute reductions in rates of aggression from 12% to 2% in up to 2 years of follow-up.^8^ We used harder outcomes, a longer follow-up, and a much larger sample size than did these trials (panel). Moreover, our data suggest that mood stabilisers could be used in a different way in clinical practice. As sole pharmacological agents, trial evidence has been ambiguous as to whether mood stabilisers reduce aggression or violence^24^, ^42^ with no positive effect recorded in higher quality studies.^42^ We report that people with bipolar disorder who take mood stabilisers have a reduced risk of committing violent crime, but these potential effects of mood stabilisers were not demonstrated in people with schizophrenia or related psychoses.

Panel **Research in context**

**Systematic review**

We searched PubMed for published studies about the violence-reducing effects of antipsychotics and mood stabilisers with the term “(antipsychotic* OR antimanic* OR mood stabiliser OR mood stabilizer OR antiepileptic*) AND (aggression OR violence OR crime OR offending)” from Jan 1, 1966, up to Nov 28, 2013. We did not use any language or date restrictions, and we used “clinical trial”, “journal article”, “review”, and “humans” as filters. The search yielded 1310 publications; of particular interest were seven recent systematic reviews about adults,^8^, ^12^, ^24^, ^42^, ^43^, ^44^, ^45^ three about children and adolescents,^46^, ^47^, ^48^ and one multi-group randomised controlled trial of adults.^49^

Existing evidence suggests that antipsychotics^8^ and valproate^45^ might reduce aggressive acts in patients with schizophrenia, but these studies are limited to aggression outcomes. Clozapine reportedly has stronger anti-aggressive qualities than other antipsychotics.^43^ Uncertainty exists about whether first-generation or second-generation antipsychotics are more effective,^44^, ^49^ and regarding the use of depot versus oral medication in patients with schizophrenia.^50^ Antipsychotics and mood stabilisers reportedly reduced anger in some patients with borderline personality disorder.^12^ Risperidone might reduce aggression in young people to a greater extent than other antipsychotics in those with disruptive behaviour disorders^47^, ^48^ and autism spectrum disorder.^46^ In the general population, mood stabilisers might reduce aggression^24^, ^42^ but these effects have not been proven in high-quality studies.

**Interpretation**

To our knowledge, this Article is the first total population-based study to address the potential violence-reducing effects of antipsychotics and mood stabilisers with a within-individual research design. Although we cannot make direct causal interpretations, our findings suggest that the benefits of antipsychotics might include effects on various crime outcomes. This idea is consistent with the 83% relative reduction in aggression in schizophrenia reported in a recent synthesis of trial data.^8^ Furthermore, previous studies about whether pharmacological monotherapy with mood stabilisers reduces violence have been equivocal.^51^, ^52^ However, we reported that men with bipolar disorder who take mood stabilisers have a reduced rate of violent crime, but we recorded no such associations with mood stabilisers in patients with schizophrenia and related disorders. Our findings need additional confirmation in clinical trials and experimental studies but might be able to help management decisions for patients with psychiatric disorders.

Second, the addition of antipsychotics to mood stabilisers was more effective than was a mood stabiliser alone, but the addition of a mood stabiliser to an antipsychotic did not have any effect. In other words, for the patient with schizophrenia, the addition of a mood stabiliser does not seem warranted to reduce violence risk. This finding is important because coadministration of antipsychotics and mood stabilisers is common in psychiatry^53^, ^54^ despite an uncertain evidence base.^25^, ^45^, ^55^ For example, one US regional survey showed that 47% of 8405 inpatients diagnosed with schizophrenia received a mood stabiliser.^56^ One concern about polypharmacy is the risk of side-effects, especially metabolic ones, in patients with schizophrenia. These patients have high rates of all-cause mortality compared with the general population—a situation that seems to have worsened in recent decades.^14^ By contrast, if concerns exist about violence risk in a patient with bipolar disorder, the addition of an antipsychotic could be considered.

Third and finally, our results suggest that associations exist between reductions in rates of violence and the use of depot antipsychotic drugs, and that this relation is at least as strong as for orally administered antipsychotics. The known benefits of depot drugs for prevention of relapse^28^ have not been extended to include other outcomes.^26^ The potential reduction on violence risk will need to considered alongside less scope to titrate the dose, and low tolerability for some patients.^57^

Our research has some limitations. First, we did not investigate how the associations between medication and violent crime are mediated, and therefore we cannot assert causal effects from our findings. Antipsychotic or mood-stabilising medication might lead to non-pharmacological benefits, such as more regular contact with health-care staff, psychological interventions, or support from family and carers, and these indirect effects might be what are actually being measured in our results. However, this limitation would not be consistent with the differences reported by diagnosis and type of medication. For example, the indirect effects of medication (such as increased support) would also be expected in those with schizophrenia who received mood stabilisers, in whom we did not record a significant rate reduction. Furthermore, we studied the effects of being prescribed another oral drug, a selective serotonin reuptake inhibitor antidepressant, in this particular cohort and recorded no reduction in violence risk. A related threat to validity is reverse causality—that is, individuals who intend to commit crimes could stop taking their prescribed medication as part of a series of psychosocial changes that predate their crime, or they might receive treatment after a violent crime. However, this possibility would not be consistent with the analysis on the timing of medication (being on medication and then off, versus first being off medication and then on) that led to similar results. If reverse causality was a major explanation, the latter pattern would not be significant. Additionally, since we included all violent crimes per individual, reverse causality would more clearly apply to those who committed only one violent crime during follow-up.

Another limitation is that confounding by indication can theoretically also occur between classes of psychotropic medication. For example, changes from antipsychotics to mood stabilisers and vice versa might occur depending on clinical severity, individual response to treatment, and comorbidity. Moreover, our data are not sensitive enough to investigate disease phase (prodromal, acute, or chronic), which is an important area for future work. A further limitation is that we used violent conviction as our primary outcome. Violent conviction does not capture the violence that less often leads to arrest, including minor violence towards family members, social care and health-care professionals, and possibly other vulnerable people. Furthermore, the date of criminal behaviour based on conviction might have led to misclassification because an individual could have been behaving antisocially before this date. However, the use of violent convictions as an outcome is more generalisable than informant or self-reported violence, and represents a higher public health burden to the individual and society in terms of costs, stigma, and possibly disease course than these other outcomes. Nevertheless, we also used data from the suspicions register to test the possibility that patients prescribed psychotropic medications might have their charges dropped more often than those without medications, and we did not record any noteworthy differences in the effects of medication on violent crime. Although our results were underpowered to assess clozapine, we did note a rate reduction using the suspicions register. Moreover, the analyses were based on dispensed prescriptions for medication (ie, those picked up by the individuals themselves, family members, or health-care staff) and we cannot be certain that these medications were actually taken by patients as intended (apart from the depots). However, this problem is similar to that in randomised controlled trials, in which intention-to-treat analyses are mainly reported. The fact that some patients in our study probably did not take their medication as prescribed would reduce the drugs' possible effects on violent crime. Hence, we believe that our findings might be conservative estimates of the actual effects of medication. One way to assess non-adherence was to compare the first 45 days after the start of treatment (when one would expect better adherence rates) with subsequent time (>45 days after initiation of treatment); however, we noted little difference in rates of violence reduction by timing of medication initiation. Finally, we did not study polypharmacy beyond combination treatment of antipsychotics and mood stabilisers, and possible differences in individual antipsychotics and mood stabilisers need further analysis.^49^

We note that only about 40% of patients taking antipsychotics and mood stabilisers had a diagnosis of schizophrenia, other psychosis, or bipolar disorder, which means that these medications are used widely for other diagnostic groups and for patients without any formal psychiatric diagnosis, and could explain the recent large increases in their use.^1^ Nevertheless, the consistency of our findings across diagnostic boundaries suggests the mechanism of the antipsychotic action in risk reduction might not only include psychotic symptoms, but also behavioural traits of anger and hostility.^58^ Furthermore, mood stabilisers might act on mood instability, which are prominent in bipolar disorder and personality disorder, but also extend to subclinical problems.

An important issue is how generalisable our findings are to other countries. For example, according to data from the US Food and Drug Administration, monthly rates of antipsychotic prescriptions in US adults (>25 years of age) were 700–850 per 100 000 population in 2004–06.^59^ This rate is not dissimilar to that in Sweden in 2006 of 760 per 100 000 population. Although self-reported crime victimisation rates have been decreasing internationally, the UK, USA, and Sweden have fairly similar rates of violent crimes reported to the police, including assault, robbery, and rape, whereas rates of the most serious offences (such as homicide) are substantially higher in the USA.^60^ In 2006, rates of assault recorded by the police in Sweden were 845 per 100 000 people compared with 787 per 100 000 in the USA.^60^ In England and Wales, the rate was higher than in Sweden at 1365 per 100 000 population in 2006.^60^

In summary, in this large population-wide study, we recorded reductions in violent crime in patients who were prescribed antipsychotics. Rates of violent crime were also reduced in patients with bipolar disorder who were receiving mood stabilisers. Therefore, in addition to the effects of antipsychotics and mood stabilisers on relapse rates, their potential effects on violence and crime should also be taken into account in decisions about management for these groups of patients.
