# Supplementary material for: Antipsychotics, mood stabilisers, and risk of violent crime
Source: Lancet. 2014 Sep 27;384(9949):1206–14. doi: 10.1016/S0140-6736(14)60379-2 (PMC4165625; doi:10.1016/S0140-6736(14)60379-2)
Supplement: Supplementary appendix [file mmc1.pdf]

THE LANCET

Supplementary appendix

This appendix formed part of the original submission and has been peer reviewed. We post it as supplied by the authors.

Supplement to: Fazel S, Zetterqvist J, Larsson H, Långström N, Lichtenstein P. Antipsychotics, mood stabilisers, and risk of violent crime. *Lancet* 2014; published online May 8. http://dx.doi.org/10.1016/S0140-6736(14)60379-2.

Web appendix

Appendix extra methods

Diagnostic codes

Schizophrenia was defined as having received at least two diagnoses of the following: ICD-10: F20; ICD-9: 295A–G, 295W, 295X; ICD-8: 295.0–6, 295.8, 295.9. Bipolar disorder (ICD-10: codes F30-F31; ICD-9: 296A, 296C-296E, 296W, 296X; ICD-8: 296.1, 296.3, 296.8, 296.9) was also defined as having at least two diagnoses, but excluding individuals defined as having schizophrenia as above. Other psychoses were defined as having at least one diagnosis (ICD-10: F10.5, F11.5, F12.5, F13.5, F14.5, F15.5, F16.5, F17.5, F18.5, F19.5, F21-F25, F28-F29, F32.3 and F33.3; ICD-9: 291, 292, 297-299, 295H or 296B; ICD-8: 291, 297, 298, 294.3 or 296.2), but excluding individuals with any episode of schizophrenia or bipolar disorder. Finally, non-psychotic depression and related mood disorders included those with at least one diagnosis (ICD-10: F32-F39 excluding F32.3 and F33.3 [psychotic depression]; ICD-9: 311, 300E; ICD-8: 300.4), and who did not have any diagnosis of schizophrenia, bipolar disorder, or other psychosis.

Timing effects

To examine whether the association between medication and rate of violent crime might be confounded by other factors associated with start of medical treatment, we studied the odds of convicted violent crimes at start of medical treatment and at the end of medical treatment. This was done by comparing pairs of contiguous time periods (45 days each) on and off treatment either in direct connection with start of treatment or with end of treatment. Treatment was defined as treatment with mood stabilizers, antipsychotics or clozapine. The outcome was convicted violent crime during the time period and exposure was treatment status. Thus, each comparison represented either a treatment start or a treatment termination. The data were then analyzed with conditional logistic regression with each pair of contiguous time periods entering a separate stratum. The analysis was done separately for treatment starts and treatment ends.

Dose response effects

Dose response effects were investigated by classifying the medication dose in terms of the defined daily dose (DDD). The dose was stratified into either 0 (no medication), between 0 and 1, between 1 and 2, or greater than 2 DDDs. Since precise dosing information was not available, the dose between two dispensing dates was estimated by dividing the amount of the medication collected the first date with the number of days between the two dates. This was analyzed with stratified Cox regression where each medication group was coded with a categorical covariate for the dose. For each medication group, the hypothesis that the effect was the same for all non-zero doses was tested against the hypothesis of greater protective effects with greater dose, using the chi-bar-square statistic by Silvapulle and Sen for joint tests with inequality constraints (ref: Silvapulle MJ and Sen PK, ***Constrained Statistical Inference: Order, Inequality, and Shape Constraints***, New York: John Wiley & Sons, 2004). More specifically, the joint alternative hypothesis was that the effect of doses between 1 and 2 is stronger than the effect of doses between 0 and 1 and that the effect of doses greater than 2 is stronger than the effect of doses between 1 and 2 (indicating greater protective effect with higher dose).

Adherence effects
Since adherence can be expected to be better during the beginning of the treatment, the treatment time was split up into the first 45 days of treatment and the remaining treatment time. The first 45 days of treatment were coded with one binary covariate and the remaining days of treatment were coded with another binary covariate. This was done for mood stabilizers, antipsychotics and clozapine separately and the data were then analyzed with stratified Cox regression model. The estimated parameters were then tested for equality for each of the medication groups with a Wald chi-square test.

Etable 1. Hazard ratios (HR) for violent crime in men and women stratified by age and hospitalization status who were prescribed antipsychotics and mood stabilizers (within-individual analyses)

**Cohort**	**No. of individuals in the study cohort**	**No. of crimes in study cohort**	**Mood stabilizer HR (95% CI)**	**Antipsychotic HR (95% CI)**	**Clozapine HR (95% CI)**
All	82,647	4,948	0.75 (0.61-0.93)	0.56 (0.48-0.65)	0.53 (0.16-1.74)
Censoring hospitalization periods	71,652	3,900	0.76 (0.60-0.97)	0.49 (0.41-0.59)	0.35 (0.03-3.69)
Age at start of follow-up					
15-24 years	21,084	2,041	1.02 (0.73-1.43)	0.59 (0.46-0.75)	-
25-39 years	42,420	2,192	0.69 (0.50-0.94)	0.53 (0.42-0.67)	-
≥40 years	19,143	715	0.49 (0.30-0.80)	0.52 (0.34-0.79)	1.26 (0.29-5.39)

Etable 2. Hazard ratios (HR) for violent crime in individuals with different psychiatric disorders who have been prescribed antipsychotics and mood stabilizers (within-individual analyses) by criminal history.

	Not convicted of violent crime before 1 January 2006			Convicted of violent crime before 1 January 2006		
**Patient groups**	**No. of individuals (No. of crimes)**	**Antipsychotic HR (95% CI)**	**Mood stabilizer HR (95% CI)**	**No. of individuals (No. of crimes)**	**Antipsychotic HR (95% CI)**	**Mood stabilizer HR (95% CI)**
Individuals ever prescribed both mood stabilizer and antipsychotic during follow-up	73,265 (2,079)	0.52 (0.41-0.66)	0.71 (0.52-0.97)	9,382 (2,869)	0.59 (0.48-0.72)	0.79 (0.60-1.05)
Any psychotic Disorder	28,845 (1,000)	0.40 (0.29-0.56)	0.58 (0.35-0.95)	5,333 (1,445)	0.58 (0.45-0.74)	0.73 (0.49-1.10)
Schizophrenia	7,896 (248)	0.48 (0.26-0.89)	1.12 (0.30-4.25)	2,339 (447)	0.81 (0.51-1.27)	1.39 (0.44-4.36)
Bipolar disorder	13,176 (344)	0.29 (0.12-0.73)	0.38 (0.19-0.77)	1,028 (221)	0.89 (0.40-1.98)	0.50 (0.27-0.92)
Other psychotic disorders	10,834 (516)	0.38 (0.24-0.59)	0.99 (0.39-2.53)	2,301 (893)	0.45 (0.33-0.63)	0.88 (0.47-1.65)
Depression	12,846 (413)	0.97 (0.54-1.75)	1.04 (0.50-2.17)	1,448 (435)	0.63 (0.36-1.11)	0.81 (0.40-1.67)

ETable 3. Hazard ratios (HR) for the association between convicted violent crime and different medication doses in a cohort of individuals prescribed mood stabilizers, antipsychotics, and clozapine compared to periods when these same individuals are without the respective medication (within-individual estimates)

	**HR (95% CI)**			
Medication group	No medication	Dose between 0 and 1 DDD per day	Dose between 1 and 2 DDDs per day	Dose more than 2 DDDs per day
Mood stabilizers	1 (ref)	0.76 (0.60-0.96)	0.51 (0.33-0.79)	0.71 (0.38-1.31)
Antipsychotics	1 (ref)	0.57 (0.48-0.69)	0.51 (0.38-0.70)	0.32 (0.19-0.51)
Clozapine	1 (ref)	0.92 (0.22-3.76)	0.31 (0.06-1.62)	-

ETable 4. Hazard ratios (HR) for the association between convicted violent crime and medication stratified by time since start of treatment in a cohort of individuals prescribed mood stabilizers, antipsychotics, and clozapine compared to periods when these same individuals are without the respective medication (within-individual estimates)

	**HR (95% CI)**		
Medication group	No medication	Short-term effect (first 45 days of medication)	Long-term effect (>45 days since treatment start)
Mood stabilizers	1 (ref)	0.76 (0.60-0.96)	0.51 (0.33-0.79)
Antipsychotics	1 (ref)	0.57 (0.48-0.69)	0.51 (0.38-0.70)
Clozapine	1 (ref)	0.92 (0.22-3.76)	0.31 (0.06-1.62)

ETable 5. Hazard ratios (HR) for violent crime in men and women with different psychiatric disorders who were prescribed antipsychotics and mood stabilizers (within-individual analyses)

	Males			Females		
**Diagnostic subgroup**	**No. of individuals (No. of crimes)**	**Antipsychotic (HR, 95% CI)**	**Mood stabilizer (HR, 95% CI)**	**No. of individuals (No. of crimes)**	**Antipsychotic (HR, 95% CI)**	**Mood stabilizer (HR, 95% CI)**
Any psychotic Disorder	17,532 (2,033)	0.53 (0.43-0.66)	0.69 (0.50-0.96)	16,646 (412)	0.38 (0.23-0.63)	0.43 (0.17-1.14)
Schizophrenia	6,015 (455)	0.69 (0.46-1.02)	1.50 (0.61-3.70)	3,110 (87)	0.50 (0.17-1.43)	*-
Bipolar disorder	4,303 (379)	0.72 (0.38-1.36)	0.44 (0.27-0.72)	7,615 (115)	0.10 (0.01-0.78)	0.49 (0.13-1.80)
Other psychotic disorders	7,214 (1,199)	0.42 (0.32-0.57)	0.94 (0.54-1.62)	5,921 (210)	0.44 (0.23-0.82)	0.67 (0.12-3.68)
Depression	5,731 (392)	0.74 (0.47-1.16)	0.86 (0.47-1.56)	8,563 (156)	0.91 (0.34-2.43)	1.06 (0.39-2.93)

Note: All analyses adjusted for clozapine medication. 'Any psychotic disorder' includes schizophrenia, bipolar disorder, and other psychotic disorders. 'Other psychotic disorders' excludes schizophrenia and bipolar disorder.
* No estimate possible due to very low base rates.

ETable 6. Associations between violent crime and different psychotropic medication exposures in men and women.

		Between-individual estimates		Within-individual estimates	
		HR (95% CI)		HR (95% CI)	
**Exposure**	**Cohort**	**Males**	**Females**	**Males**	**Females**
Adding an antipsychotic during periods on mood stabilizers	Individuals ever prescribed both mood stabilizer and antipsychotic during follow-up	0.72 (0.49-1.04)	0.61 (0.32-1.18)	0.25 (0.07-0.87)	0.33 (0.04-3.21)
Adding a mood stabilizer during periods on antipsychotics	Individuals ever prescribed both mood stabilizer and antipsychotic during follow-up	0.77 (0.54-1.08)	0.65 (0.33-1.26)	0.82 (0.32-2.13)	0.50 (0.05-5.51)
Antipsychotic (AP) depot	Prescribed depot AP during follow-up	0.37 (0.28-0.49)	0.40 (0.25-0.66)	0.67 (0.43-1.06)	0.67 (0.27-1.67)
Antipsychotic (AP) depot*	Prescribed depot AP during follow-up	0.30 (0.22-0.40)	0.30 (0.19-0.49)	0.62 (0.39-0.99)	0.51 (0.19-1.39)
Antipsychotic (AP) oral medication	Prescribed oral AP during follow-up	0.39 (0.35-0.44)	0.58 (0.46-0.74)	0.55 (0.46-0.66)	0.43 (0.29-0.65)
SSRI medication	Prescribed mood stabilizer, AP or clozapine during follow-up	0.91 (0.81-1.02)	1.14 (0.92-1.4)	1.09 (0.89-1.33)	1.41 (0.94-2.16)

Note: * adjusted for oral antipsychotics, mood stabilizers, and clozapine.

EFigure 1. Extended Kaplan-Meier curves comparing time on medication (solid lines) to time off medication (dotted lines): (a) Mood stabilizer, antipsychotic or clozapine vs no medication, (b) Mood stabilizer vs no mood stabilizer, (c) Antipsychotics vs no antipsychotics, and (d) Clozapine vs no Clozapine. All individuals in this cohort had at least one prescription of mood stabilizer, antipsychotic, or Clozapine during follow up. For (b)-(d), adjustments are not made for other medications.

a b

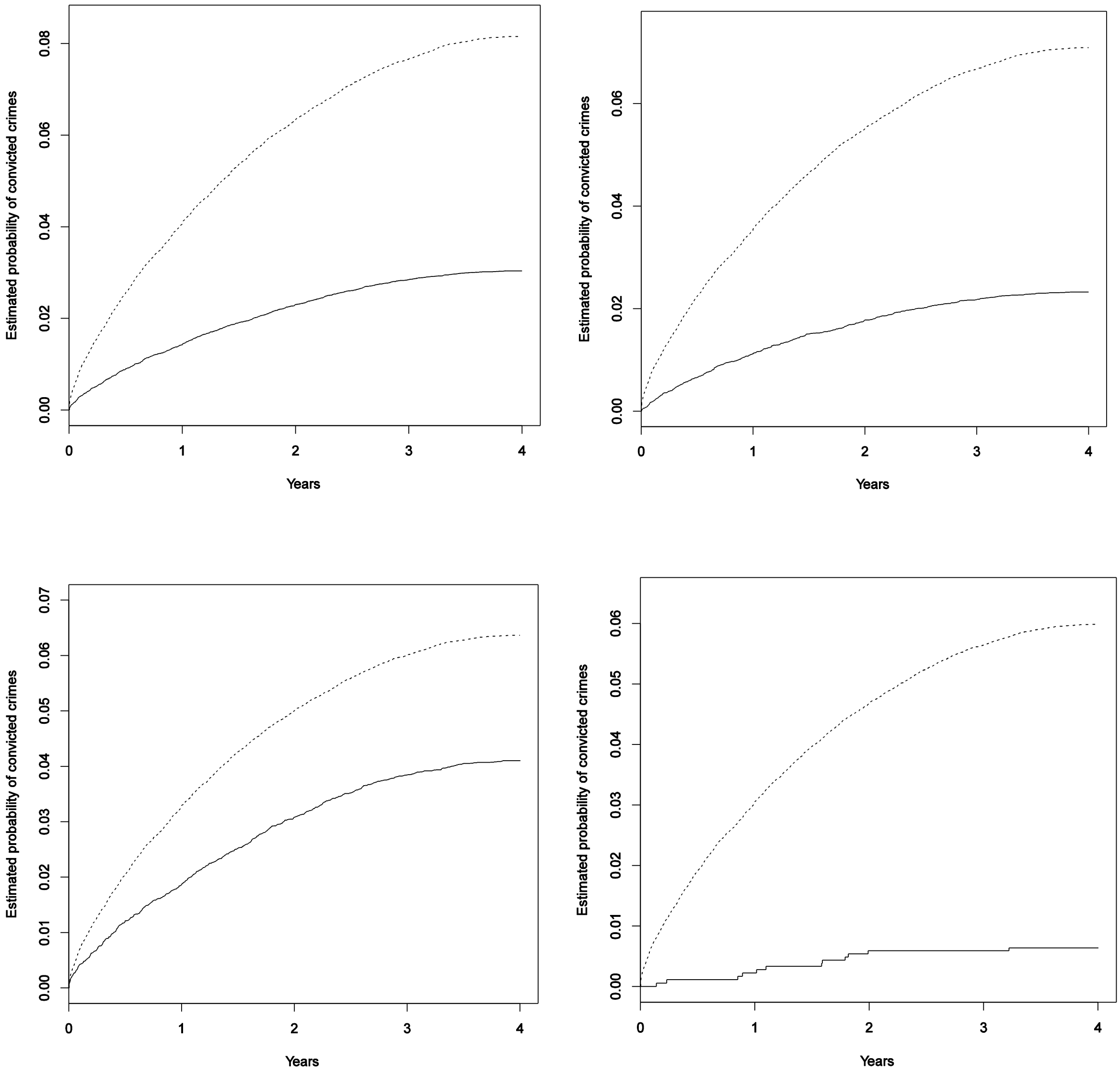

c d
